# Altered Resting-State Functional Connectivity of the Frontal-Striatal Reward System in Social Anxiety Disorder

**DOI:** 10.1371/journal.pone.0125286

**Published:** 2015-04-30

**Authors:** Joshua Manning, Gretchen Reynolds, Zeynep M. Saygin, Stefan G. Hofmann, Mark Pollack, John D. E. Gabrieli, Susan Whitfield-Gabrieli

**Affiliations:** 1 McGovern Institute for Brain Research, Massachusetts Institute of Technology, Cambridge, Massachusetts, United States of America; 2 Poitras Center for Affective Disorders Research, Massachusetts Institute of Technology, Cambridge, Massachusetts, United States of America; 3 Department of Brain and Cognitive Sciences, Massachusetts Institute of Technology, Cambridge, Massachusetts, United States of America; 4 Department of Psychology, Boston University, Boston, Massachusetts, United States of America; 5 Rush University Medical Center, Chicago, Illinois, United States of America; Leibniz Institute for Neurobiology, GERMANY

## Abstract

We investigated differences in the intrinsic functional brain organization (functional connectivity) of the human reward system between healthy control participants and patients with social anxiety disorder. Functional connectivity was measured in the resting-state via functional magnetic resonance imaging (fMRI). 53 patients with social anxiety disorder and 33 healthy control participants underwent a 6-minute resting-state fMRI scan. Functional connectivity of the reward system was analyzed by calculating whole-brain temporal correlations with a bilateral nucleus accumbens seed and a ventromedial prefrontal cortex seed. Patients with social anxiety disorder, relative to the control group, had (1) decreased functional connectivity between the nucleus accumbens seed and other regions associated with reward, including ventromedial prefrontal cortex; (2) decreased functional connectivity between the ventromedial prefrontal cortex seed and lateral prefrontal regions, including the anterior and dorsolateral prefrontal cortices; and (3) increased functional connectivity between both the nucleus accumbens seed and the ventromedial prefrontal cortex seed with more posterior brain regions, including anterior cingulate cortex. Social anxiety disorder appears to be associated with widespread differences in the functional connectivity of the reward system, including markedly decreased functional connectivity between reward regions and between reward regions and lateral prefrontal cortices, and markedly increased functional connectivity between reward regions and posterior brain regions.

## Introduction

A fundamental goal of neuroscience is to understand the functional organization of brain networks that underlie neuropsychiatric disease. One prevalent disease is social anxiety disorder (SAD) [1], a chronic psychiatric disorder characterized by fear of negative evaluation by others, which in turn leads to high levels of anxiety and avoidance of social situations [2]. Multiple neuroimaging studies have focused on an increased amygdala response that is associated with social anxiety [3]. Here we asked whether there are also differences in SAD of the intrinsic functional organization of the reward system of the brain, i.e. brain regions that are activated by the anticipation or receipt of reward [4, 5]. We measured intrinsic functional organization from resting-state fMRI that identifies neural networks as defined by regions exhibiting correlated, low-frequency fMRI signals in the absence of external stimuli [6, 7].

Animal and human research have converged to identify several brain regions that are consistently activated by reward, including the striatum, nucleus accumbens (NAcc), and ventromedial prefrontal cortical (vmPFC)/medial orbitofrontal (mOFC). The ventral striatum and NAcc are associated with reward anticipation [4, 5], whereas the vmPFC is associated with receipt of reward [5]. The vmPFC has also been shown to encode the value of reward outcomes in decision-making that requires action [8]. The vmPFC/mOFC likely plays a role in translating rewards to a representation of value [9]. We were guided by this literature to select seeds (brain locations) in the NAcc and vmPFC regions, and compare their intrinsic functional connectivity between the SAD and control groups.

Neuroimaging studies examining task-driven activations have indicated that the reward system is affected in SAD. People with SAD showed increased activation in the amygdala but decreased activation in OFC and vmPFC when anticipating negative emotional stimuli [10]. SAD patients also showed increased temporal correlations between the amygdala and vmPFC/mOFC compared to healthy participants in resting-state functional connectivity [11]. Striatal dysfunction also occurs in people with behavioral inhibition, a temperamental trait that is characterized by withdrawal from unfamiliar social situations and that might be a precursor of adult SAD [12–14]. Behaviorally inhibited adolescents have shown altered striatal activation, including in the caudate and NAcc, to anticipated rewards and altered vmPFC activation for reward outcomes [13, 14]. Deep-brain stimulation of the NAcc was shown to decrease generalized anxiety, showing a causal role for the NAcc in anxiety [15]. These results point to differences in the reward system between people who are socially anxious or behaviorally inhibited and those who are not.

Conceptually, it is plausible that SAD is associated with an altered reward circuitry. People typically find social interactions to be rewarding, but SAD patients often find such interactions to be aversive and anxiety provoking. Intrinsic functional connectivity may reflect chronic interactions among brain regions that mold large-scale functional networks. Reduced experience of social reward in SAD may be reflected by altered functional connectivity between the two major components of the reward system, which are also associated with social reward [16], the NAcc and the vmPFC. Translation of reward into reward value may involve interactions between NAcc and vmPFC, and decreased NAcc-vmPFC connectivity may reflect weakened translation of reward into reward value. We hypothesized that people with SAD would show decreased functional connectivity between the NAcc and the vmPFC.

## Methods

### Participants

Patients were recruited from the Center for Anxiety and Related Disorders at Boston University and the Center for Anxiety and Traumatic Stress Related Disorders at Massachusetts General Hospital. Control participants were recruited via advertisements from the community. Brain scans were performed at the Athinoula A. Martinos Imaging Center at the McGovern Institute for Brain Research at MIT. All participants gave written informed consent to all procedures. The procedures were approved by the Committee on the Use of Humans as Experimental Subjects at MIT, the Institutional Review Board at Boston University, and the Partners Human Research Committee at Massachusetts General Hospital.

Participants were 53 outpatients with SAD (generalized subtype; years with SAD: *M* = 16.8 years; age of onset: *M* = 13.4 years; age: *M* = 29.9 years; 44 right handed; 17 women) and 33 healthy Control participants (age: *M* = 29.4 years; 29 right handed; 14 women). The two groups did not differ significantly for mean age (Control: *M* = 29.4, *SD* = 6.22; SAD: *M* = 29.9, *SD* = 8.25; *t*(84) = .31, *p* = .76), the proportions of men and women, (χ^2^(1, N = 86) = .94, *p* = .33), or the proportions of right-handed and left-handed individuals (χ^2^(1, N = 86) = .37, *p* = .54). Patient and Control groups did not differ significantly on IQ as estimated with the American National Adult Reading Test Full Scale Intelligence Quotient [17] (Control: *M* = 118.6, *SD* = 7.22; SAD: *M* = 117.3, *SD* = 6.63, *t*(84) = 87, *p* = .39).

Patients were off concurrent psychotropic medication for at least two weeks prior to the scanning session. Diagnoses were confirmed with Structured Clinical Interviews for DSM-IV or the Anxiety Interview Schedule for DSM-IV [18]. The diagnosis and diagnostic subtype specifier was assessed with the Anxiety Disorders Interview Schedule IV Revised (ADIS-IV-R) by experienced clinicians. The severity of social anxiety was measured using the clinician-administered version of the Liebowitz Social Anxiety Scale (LSAS) [19] with a minimum LSAS score of 60 as an additional inclusion criterion (*M* = 81.85; range: 60–121).

Exclusion from the study occurred in the case of a lifetime history of bipolar disorder, schizophrenia, psychosis, delusional disorders or obsessive-compulsive disorder; an eating disorder in the past 6 months; a history of substance or alcohol abuse or dependence (other than nicotine) in the last 6 months and posttraumatic stress disorder within the past 6 months. Entry of patients with other mood or anxiety disorders was permitted if SAD was judged to be the predominant disorder. In addition to the primary diagnosis of SAD, 18 participants also met diagnostic criteria for comorbid major depression disorder and 16 participants for comorbid anxiety disorders (generalized anxiety disorder: n = 12; post traumatic stress disorder: n = 1; specific phobia: n = 7; panic disorder: 3). Both SAD and Control participants were excluded in the case of neurological disorders or serious medical illness.

No clinically relevant conditions in the Control group were permitted and the absence of these was confirmed prior to study inclusion using the Structured Clinical Interview for DSM-IV [18]. Patients scored significantly worse on both the Social Phobia and Anxiety Inventory (SPAI) [20] (Control: *M* = 36.7, *SD* = 23.76); SAD: *M* = 112.6, *SD* = 22.27), *t*(84) = 14.99, *p* < .0001, two-tailed) and the State and Trait Anxiety Inventory (STAI) [21] (Control: *M* = 30.0, *SD* = 7.01; SAD: *M* = 54.8, *SD* = 10.51, *t*(83) = 11.97, *p* < .0001, two-tailed). One SAD patient was missing the STAI score and was not included in this t-test.

### Imaging

#### Acquisition

Data were acquired using a 3-Tesla Siemens Tim Trio scanner (Siemens, Erlangen, Germany) with a 32-channel phased array whole-head coil. 3D T1-weighted magnetization prepared rapid acquisition gradient echo (MP-RAGE) anatomical images (TR = 2530 ms, TE = 3.39 ms, flip angle = 9°, 1 mm slice thickness, 1 mm^2^ in plane resolution) and one 6-minute resting state scan were collected while participants fixated on a cross (T2* weighted gradient echo TR/TE/Flip = 6000ms/30ms/90°, 67 contiguous interleaved oblique slices, voxel size: 2 X 2 X 2). The sequence included prospective acquisition correction (PACE) for head motion [22].

### Data Analysis

#### fMRI Analysis

The functional data were analyzed using SPM8 [23]. Functional images were preprocessed with realignment for motion correction, slice-time correction, artifact detection and spatial smoothing (8mm full-width-half-maximum Gaussian kernel). To address any spurious correlations in resting-state networks caused by head motion, we used the Artifact Detection Tools (ART, http://www.nitrc.org/projects/artifact_detect) to identify problematic time points during the scan. Specifically, an image was defined as an artifactual time point if the head displacement in X, Y, or Z direction was greater than .5 mm from the previous frame, or if the global mean intensity in the image was greater than 3 standard deviations from the mean image intensity for the entire resting scan. A two-sample t-test was performed using the number of artifactual time points to compare the difference in motion between the Control group and the SAD group.

CompCor was used to estimate physiological and other sources of noise [24]. Global signal regression was not used because it has been shown to bias and produce spurious negative correlations [25, 26]. Anatomical volumes were segmented into grey matter, white matter, and cerebrospinal fluid (CSF) areas and the resulting masks were eroded (one voxel erosion) to minimize partial volume effects. The temporal time series characterizing the estimated subject motion (3 rotation and 3 translation parameters, plus another 6 parameters representing their first-order temporal derivatives) and artifactual covariates (one covariate per artifactual time point consisting of 0’s everywhere and a “1” for the artifactual time point), as well as the blood oxygen level-dependent (BOLD) time series within the subject-specific white matter mask (3 first principal components) and CSF mask (3 first principal components), were used as temporal covariates and removed from the BOLD functional data using linear regression, and the resulting residual BOLD time series were band-pass filtered (0.008Hz < f < 0.083Hz).

**Fig 1:**
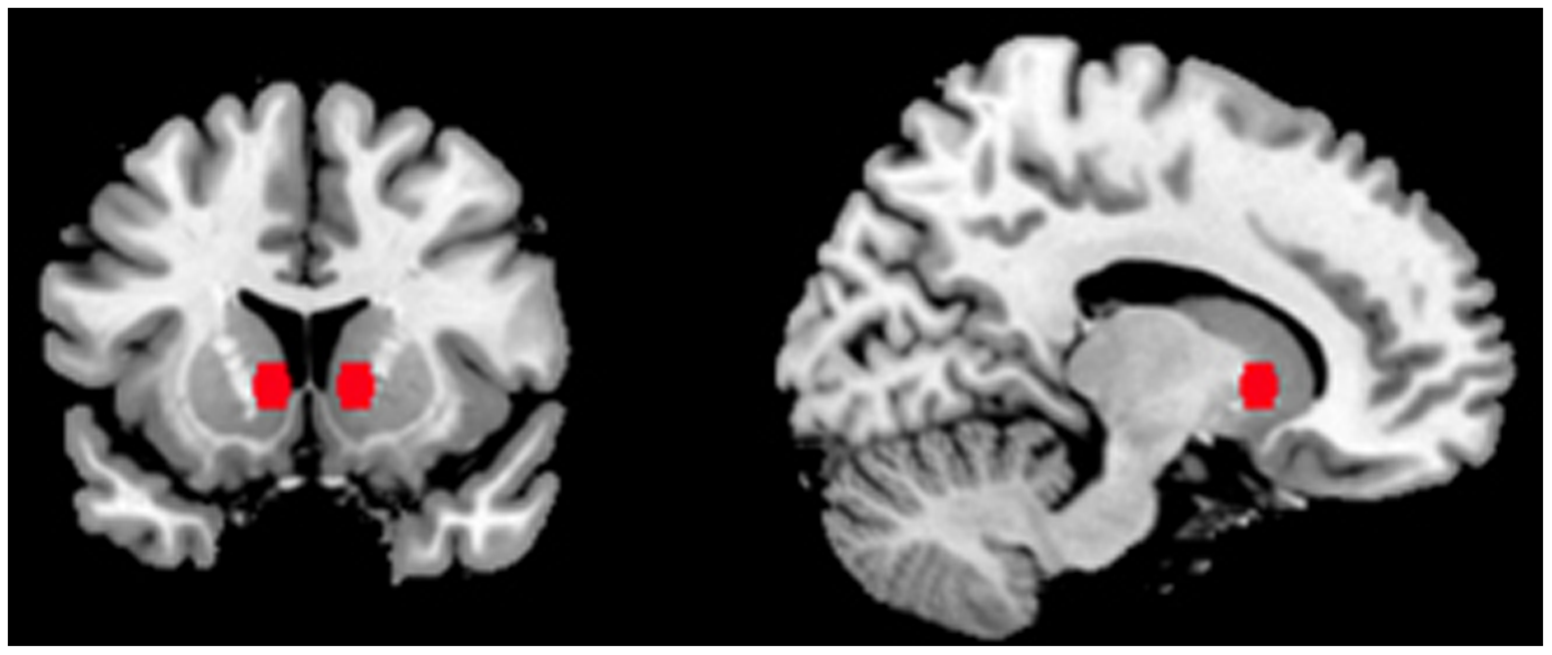
Nucleus Accumbens Seed. Bilateral nucleus accumbens seed used in resting-state functional connectivity analysis. MNI coordinates: (-8,12,1), (11,11,1).

NAcc and vmPFC were *a priori* regions of interest (ROI) to be used as seeds in the functional connectivity analysis. The seeds were selected from prior reward activation studies (and thus independent of group differences in the present study). In order to minimize the number of statistical comparison, the NAcc (left and right NAcc) and vmPFC seeds (three locations) respectively, were combined into two analyses. Two 5mm-radius ROIs, one on the left and one on the right, from the center of activation in the NAcc regions (MNI: (-8,12,1), (11,11,1); Fig 1) were selected from areas that showed increased activation to reward anticipation from Knutson *et al*. [5]. Three 10mm-radius ROIs from the center of activation in the vmPFC (MNI: (-6,24,-21), (6,30,-9), (9,27,-12)) were selected from areas that showed increased activation to reward value from Gläscher *et al*. [8]. The NAcc seeds were smaller in volume because the NAcc is considerably smaller in volume than the vmPFC.

The left and right NAcc ROIs both showed increased activation to reward anticipation simultaneously in the same study [5] and therefore were combined to create one NAcc seed (S1 and S2 Figs for analysis of separate ROIs). Similarly, the three vmPFC ROIs showed activation to reward value simultaneously in the same study [8] and therefore were combined to create one vmPFC seed (S3 and S4 Figs for analysis of separate ROIs). Consistent with activation literature, both the individual NAcc locations and the individual vmPFC locations exhibited similar patterns of functional connectivity and similar alterations in the SAD group, which supported the validity of combining the individual locations into two seeds.

First-level correlation maps were produced by extracting the mean BOLD time course from each seed and computing Pearson’s correlation coefficients between that time course and the time course of all other voxels. Correlation coefficients were converted to normally distributed Z-scores using the Fisher transformation to allow for second-level General Linear Model analysis. Two sample t-tests were performed on the Fisher transformed r-maps to examine the differences in resting state functional connectivity between Control and Patient groups. One set of t-tests examined differences for the bilateral NAcc seeds combined into a single network, and the other set of t-tests examined differences for the three vmPFC seeds combined into a single network. All results were reported using a cluster-wise false discovery rate (Cluster-wise FDR corrected) threshold of .05 for multiple comparisons. All analyses were performed in the volume and the results were projected to an inflated cortical surface for visualization.

## Results

To ensure that results were not due to motion artifacts, we compared the number of artifactual time points in the SAD and Control groups. There was no significant difference between groups in the number of artifactual time points (p = .35).

### Nucleus Accumbens Seed

#### Contrast: Control group > SAD group

**Fig 2:**
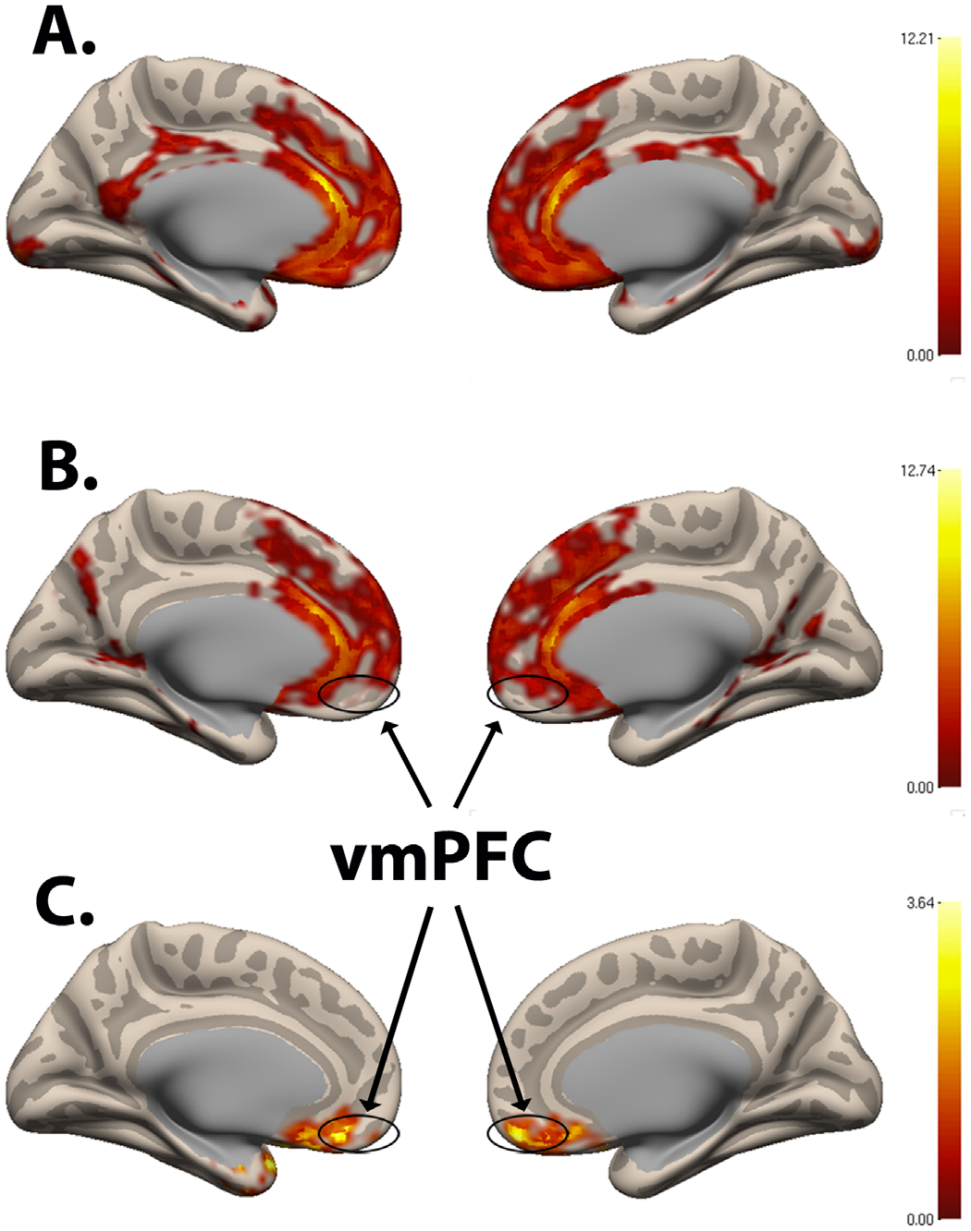
Nucleus Accumbens Seed Functional Connectivity Network of Control > SAD. Resting-state connectivity for **(A)** Control group, **(B)** SAD group, and **(C)** Control group > SAD group with bilateral nucleus accumbens seed from Fig 1 (Cluster-wise FDR corrected, p < .05; Peak voxel: (MNI Coordinates) (30, 12, -6; p = .001). (vmPFC = ventromedial prefrontal cortex).

**Fig 3:**
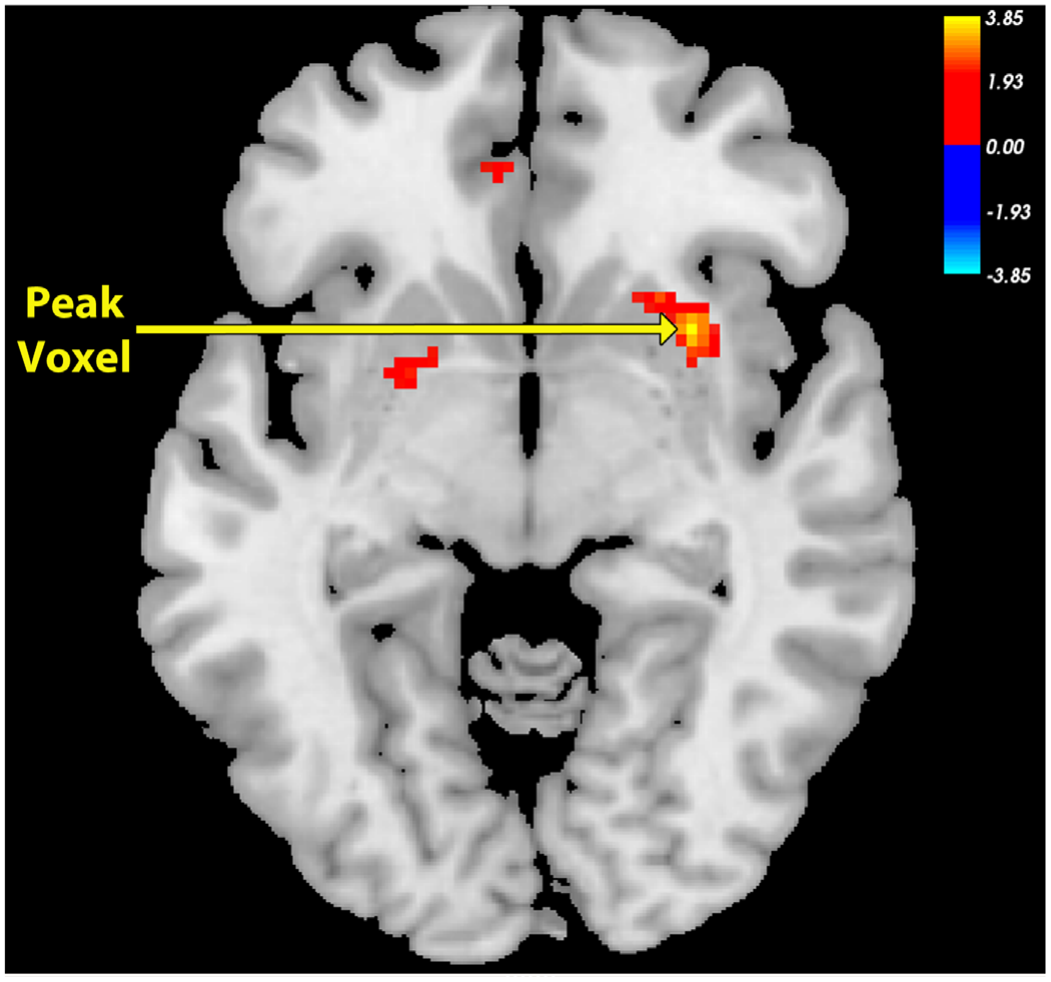
Peak Voxel Axial View for Nucleus Accumbens Seed Functional Connectivity Network of Control > SAD. Resting-state connectivity for Control group > SAD group with bilateral nucleus accumbens seed from Fig 1 (Cluster-wise FDR corrected, p < .05; Peak voxel: (MNI Coordinates) (30, 12, -6; p = .001). (vmPFC = ventromedial prefrontal cortex).

**Fig 4:**
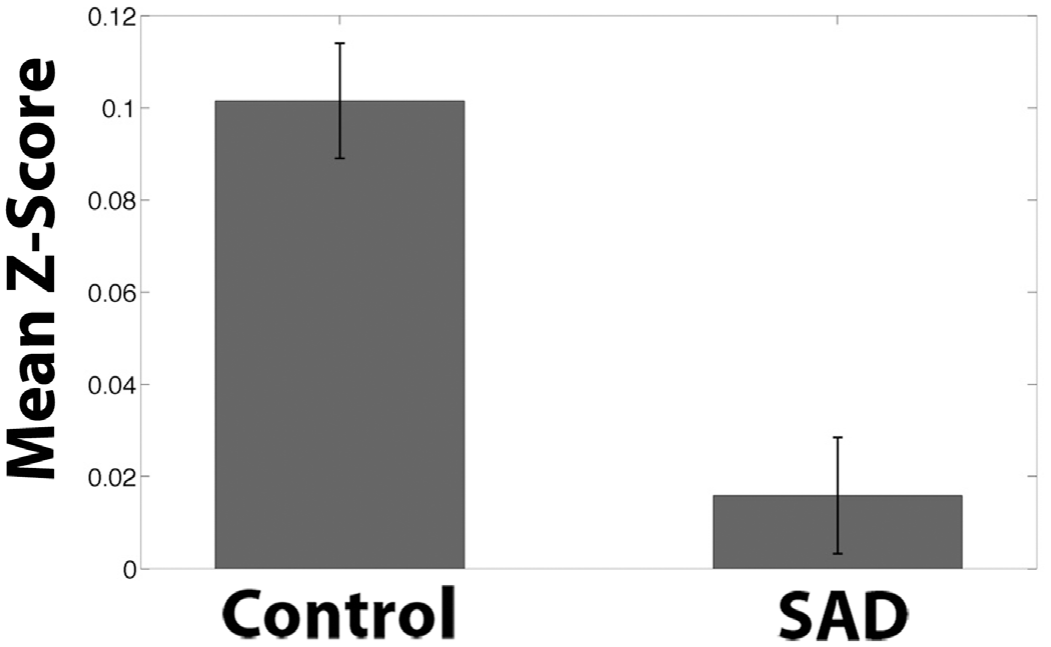
Nucleus Accumbens Seed Functional Connectivity Network Z-Scores of Control > SAD. Mean Fisher transformed correlation values for resting-state connectivity for ventromedial prefrontal cortex cluster from Fig 2C showing significant difference for Control group > SAD group with bilateral nucleus accumbens seed from Fig 1. Peak voxel (MNI coordinates): (30, 12, -6). Bars represent mean connectivity for each group for the ventromedial prefrontal cluster from Fig 2C. One-sample t-test relative to 0: Control: p < .0001; SAD: p = .22. Error bars represent standard errors.

The SAD group exhibited significant and widespread decreases in functional correlations (connectivity), compared to the Control group, between the NAcc seed (Fig 1) and multiple regions associated with reward, value, and decision-making, including vmPFC/BA11, bilateral medial anterior prefrontal cortex/BA10, bilateral inferior frontal gyrus, anterior regions of the dorsal anterior cingulate cortex (dACC), subgenual ACC, left temporal pole, left hippocampus, and bilateral putamen (Figs 2 & 3; Table 1). The region of maximal difference was a cluster that included the vmPFC, and in this cluster the Control group exhibited a significantly positive correlation, but the SAD group did not exhibit any significant correlation (Figs 2, 3 & 4).

**Table 1:** Regions of Significant Differences in Functional Connectivity Between SAD and Control Groups.

Regions for Resting State-Connectivity Clusters							
Seed	Contrast	MNI (X, Y, Z) Coordinates for Peak Voxel			Region	KE	p
**NAcc**	Control > SAD	30	12	-6	R. Putamen	1154	.001
	SAD > Control	0	-34	64	BA6	950	.006
**vmPFC**	Control > SAD: Cluster 1	-12	20	-20	BA25	1988	< .001
	SAD > Control: Cluster 1	6	-42	60	BA5	1273	.001
	SAD > Control: Cluster 2	20	-50	6	BA30	1056	.002
	SAD > Control: Cluster 3	-50	-56	-4	BA37	869	.006

MNI coordinates of peak voxels and statistical results of two-sample t-tests for functional connectivity contrasts.

To ensure that group differences were not the result of lower signal to noise ratio (SNR) in the vmPFC/mOFC cortex in the SAD group, we calculated the temporal SNR separately for the vmPFC cluster for each group. The SAD group had a higher temporal SNR in the vmPFC cluster (Control: *M* = 141.95, *SD* = 32.85; SAD: *M* = 160.81, *SD* = 46.73; *p* < .046, two-tailed). Therefore, increased functional connectivity in the Control group compared to the SAD group was not due to loss of signal in the SAD group.

#### Contrast: SAD group > Control group

**Fig 5:**
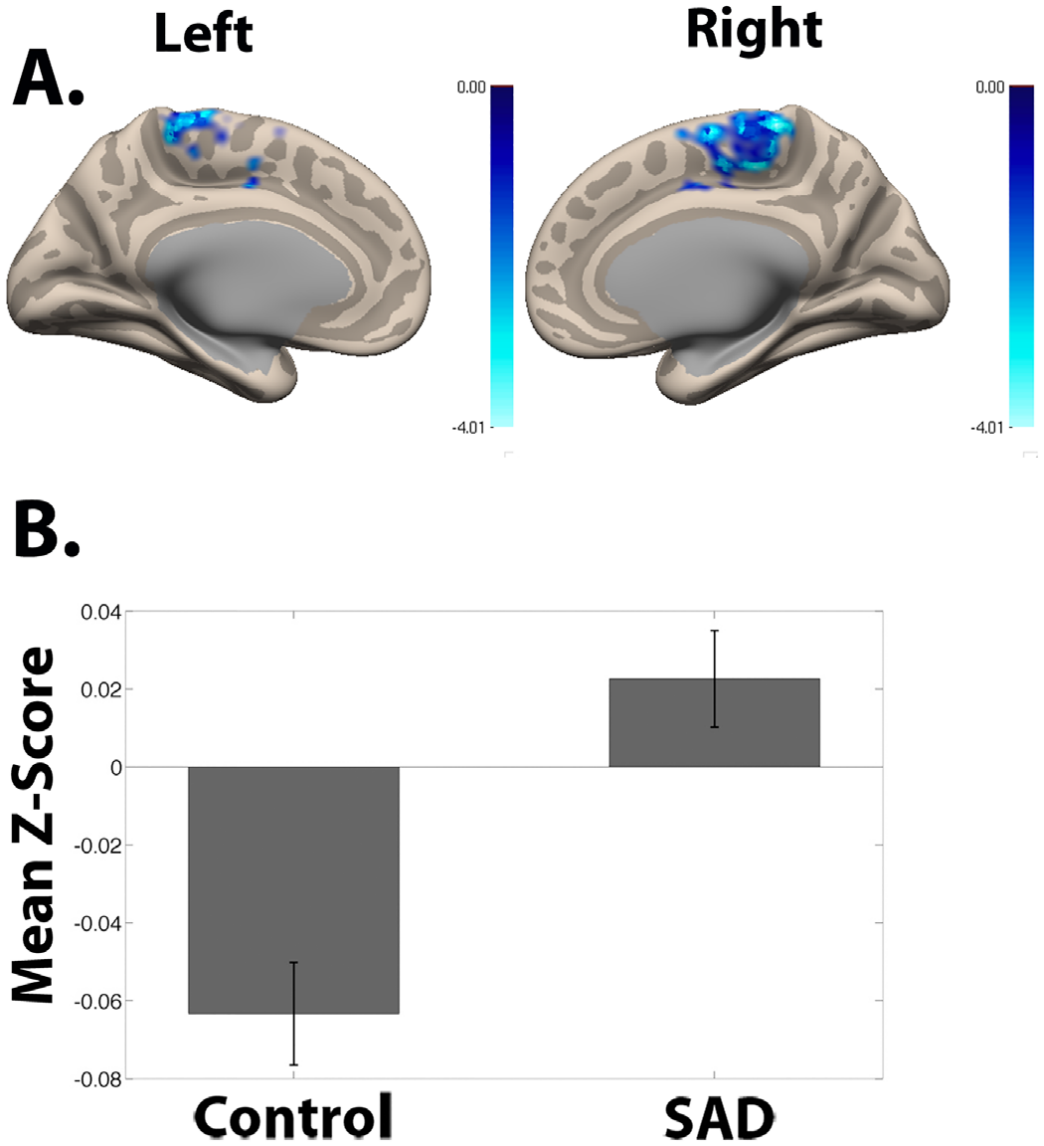
Nucleus Accumbens Seed Functional Connectivity Network of SAD > Control and Z-Scores. **(A)** Resting-state connectivity for SAD group > Control group with bilateral nucleus accumbens seed from Fig 1 (Cluster-wise FDR corrected, p < .05; Peak voxel: (MNI Coordinates) (0, -34, 64; p = .006). **(B)** Mean Fisher transformed correlation values for resting-state connectivity for cluster from Fig 5A showing significant difference for SAD group > Control group with bilateral nucleus accumbens seed from Fig 1. Peak voxel (MNI coordinates): (0, -34, 64). Bars represent the mean connectivity among for each group for the ventromedial prefrontal cluster from Fig 5A. One-sample t-test relative to 0: Control: p < .0001; SAD: p = .07. Error bars represent standard errors.

The SAD group exhibited significant increases in connectivity, compared to the Control group, between the NAcc seed and more posterior regions including bilateral somatosensory association cortex, bilateral premotor cortex, bilateral primary motor cortex, and posterior ventral ACC (vACC) (Fig 5A; Table 1). The group differences reflected a pattern of negative correlations (anticorrelations) between the NAcc seed and these posterior regions in the Control group versus a pattern of positive correlations between the NAcc seed and these regions in the SAD group (Fig 5B).

### Ventromedial Prefrontal Cortex Seed

#### Contrast: Control group > SAD group

**Fig 6:**
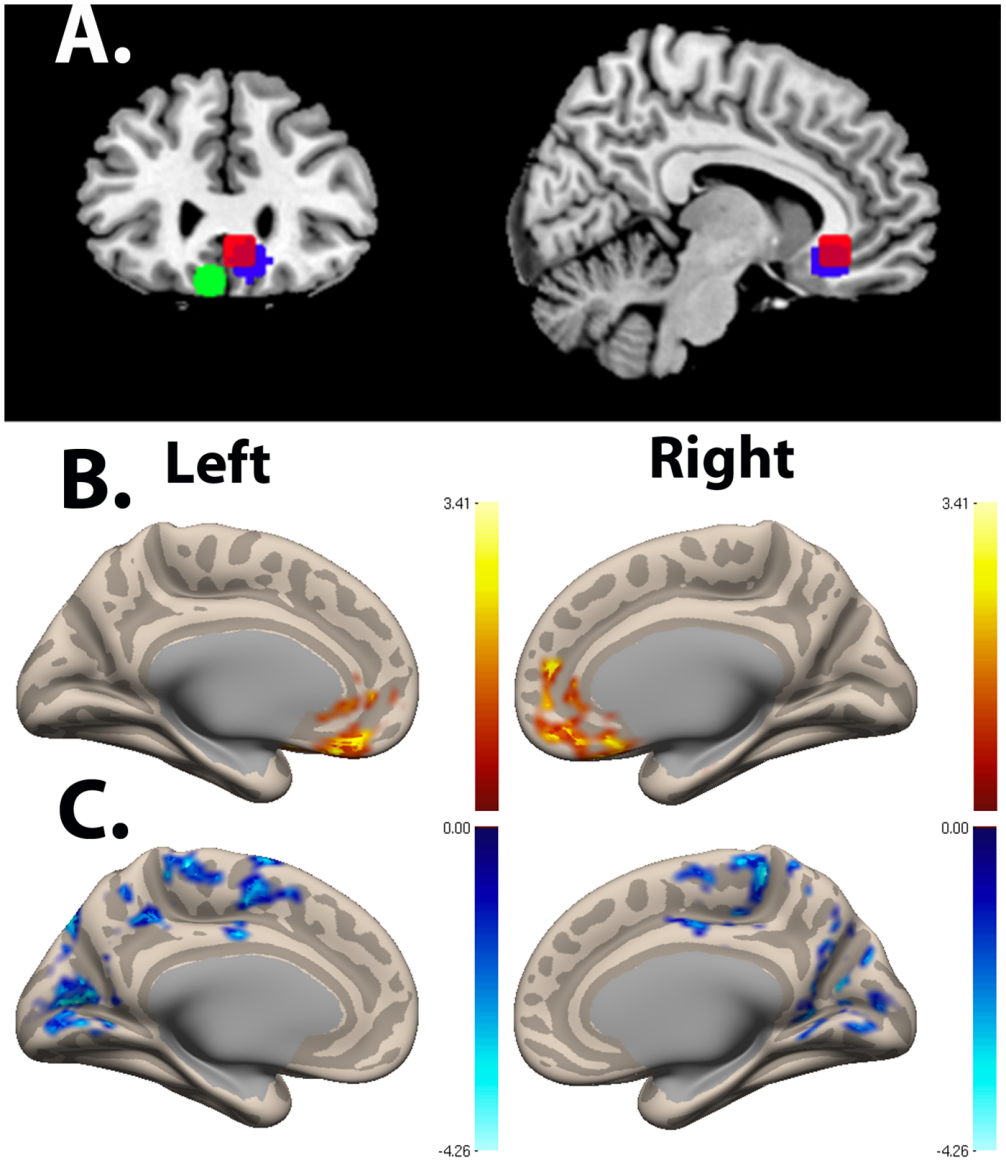
Ventromedial Prefrontal Cortex Seed Functional Connectivity Network. **(A)** Ventromedial prefrontal cortex seed used in resting-state functional connectivity analysis. MNI coordinates: (-6, 24, -21), (6, 30, -9), (9, 27, -12). **(B)** Resting-state connectivity for Control group > SAD group with ventromedial prefrontal cortex seed from Fig 6A (Cluster-wise FDR corrected, p < .05). Peak voxel (MNI coordinates): (-12, 20, -20; p < .001). **(C)** Resting-state connectivity for SAD group > Control group with ventromedial prefrontal cortex seed from Fig 6A (Cluster-wise FDR corrected, p < .05). Peak voxel cluster 1 (MNI coordinates): (6, -42, 60; p = .001). Peak voxel cluster 2 (MNI coordinates: (20, -50, 6; p = .002). Peak voxel cluster 3 (MNI coordinates): (-50, -56, -4; p = .006).

**Fig 7:**
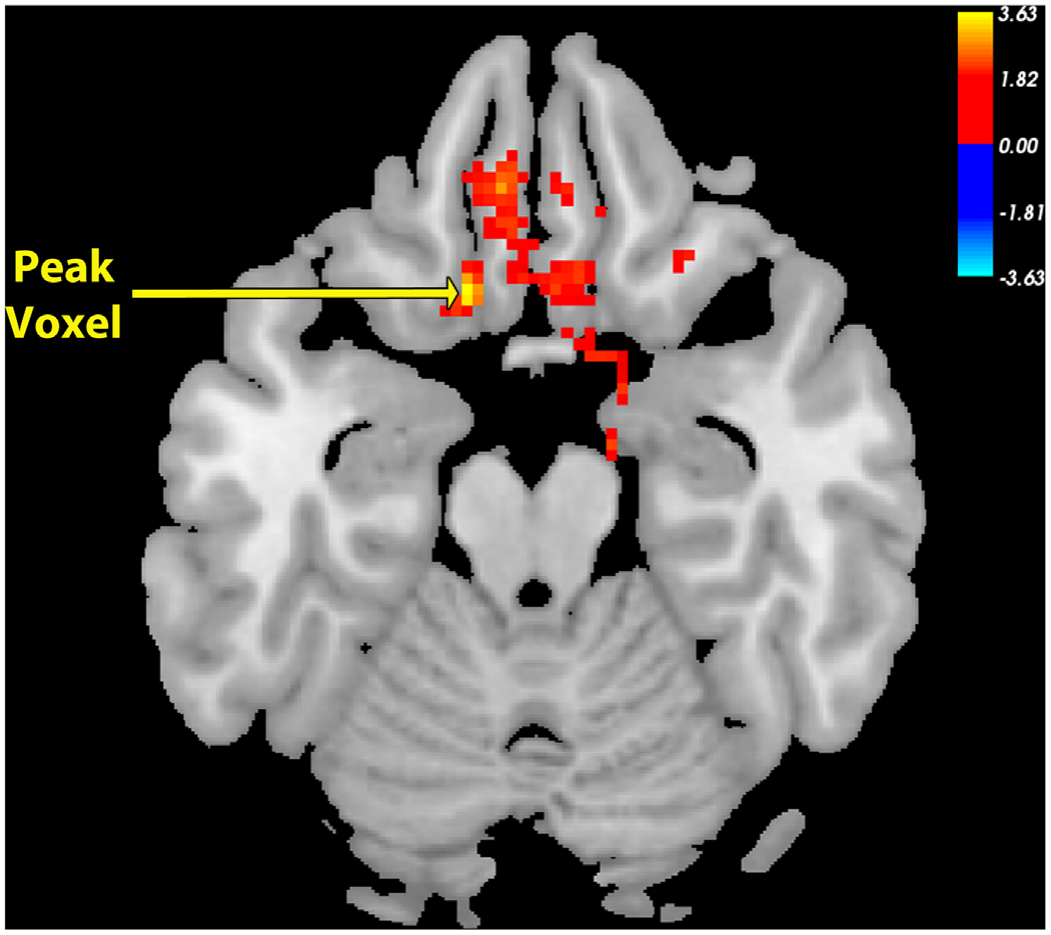
Peak Voxel Axial View for Ventromedial Prefrontal Cortex Seed Functional Connectivity Network of Control > SAD. Resting-state connectivity for Control group > SAD group with ventromedial prefrontal cortex seed from Fig 6A (Cluster-wise FDR corrected, p < .05). Peak voxel (MNI coordinates): (-12, 20, -20); p < .001).

The SAD group also exhibited significant and widespread decreases in connectivity, compared to the Control group, between the vmPFC seed (Fig 6A) and regions associated with reward, decision-making, and planning, including bilateral NAcc, bilateral medial anterior PFC/BA10, bilateral DLPFC, bilateral inferior PFC, vACC, subgenual ACC, and dACC (Figs 6B & 7; Table 1). Both groups exhibited positive correlations between the vmPFC seed and the other regions, but the correlations were significantly greater in the Control group. The region of maximal difference included the NAcc and regions associated with cognitive control, the lateral PFC. In this cluster, both the Control and SAD groups exhibited significantly positive correlations (Figs 6B, 7, & 8).

**Fig 8:**
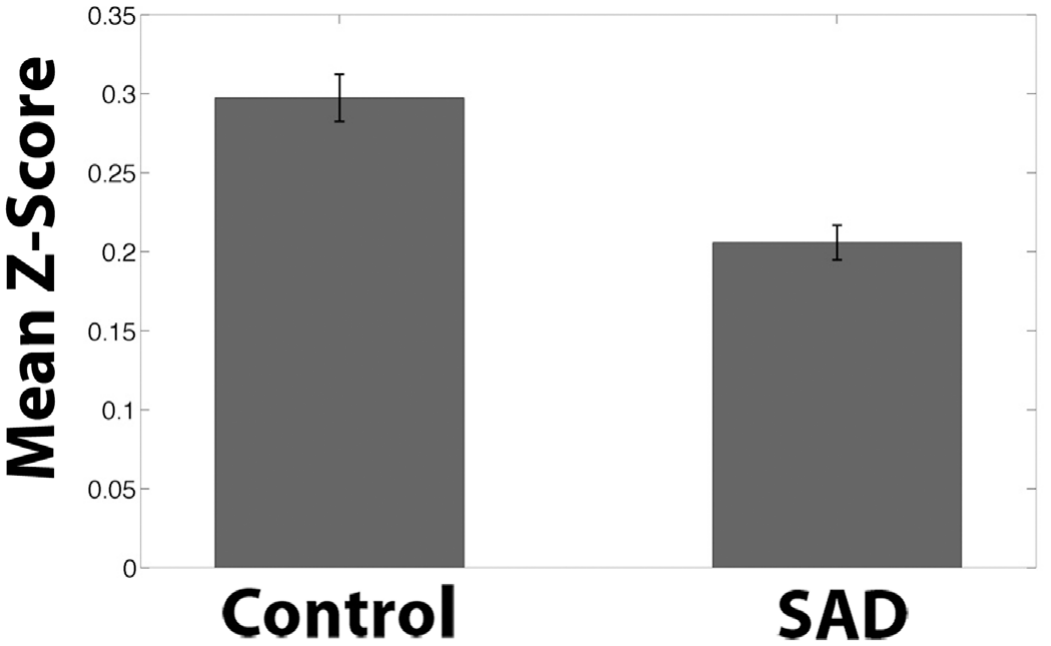
Ventromedial Prefrontal Cortex Seed Functional Connectivity Network Z-Scores of Control > SAD. Mean Fisher transformed correlation values for resting-state connectivity for clusters from Fig 5B showing significant difference for Control group > SAD group with ventromedial prefrontal cortex seed from Fig 6A. Bars represent the mean connectivity for each group for the medial prefrontal cluster from Fig 6B. Peak voxel Cluster 1 (MNI coordinates): (-12, 20, -20). One-sample t-test relative to 0: Control: p < .0001. SAD p < .0001. Error bars represent standard errors.

#### Contrast: SAD group > Control group

**Fig 9:**
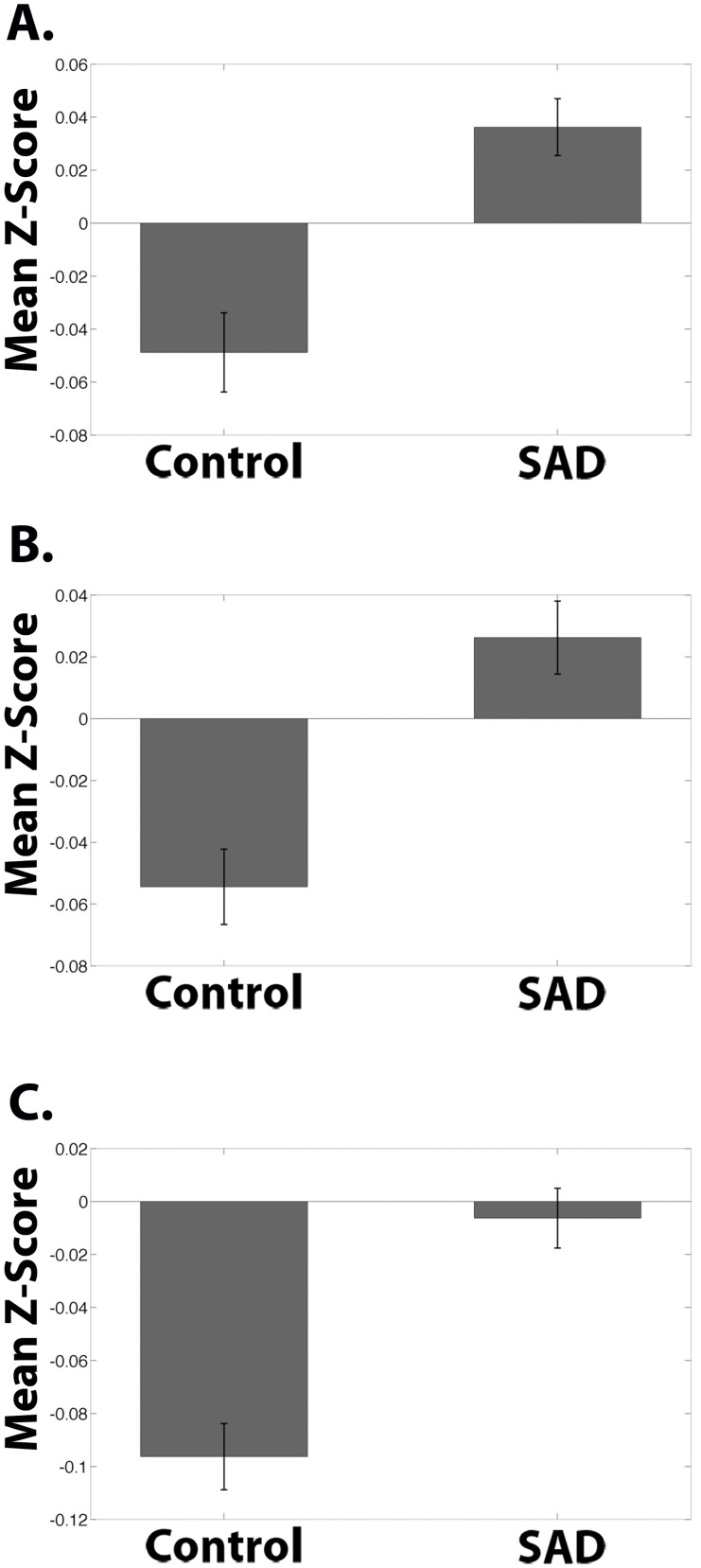
Ventromedial Prefrontal Cortex Seed Functional Connectivity Network Z-Scores of SAD > Control. Mean Fisher transformed correlation values for resting-state connectivity for clusters from Fig 6C showing significant difference for SAD group > Control group with ventromedial prefrontal cortex seed from Fig 6A. Bars represent the mean connectivity for each group for the clusters from Fig 6C. **(A)** Peak voxel Cluster 1 (MNI coordinates): (6, -42, 60). One-sample t-test relative to 0: Control: p = .003; SAD: p = .001. **(B)** Peak voxel Cluster 2 (MNI coordinates): (20, -50, 6). One-sample t-test relative to 0: Control: p < .0001; SAD: p .031. **(C)** Peak voxel Cluster 3 (MNI coordinates): (-50, -56, -4). One-sample t-test relative to 0: Control: p < .0001; SAD: p = .58. Error bars represent standard errors.

The SAD group exhibited significant increases in connectivity, compared to the Control group, between the vmPFC seed and multiple brain regions, including bilateral premotor, bilateral primary motor cortex, posterior vACC, dorsal PCC (dPCC), and bilateral somatosensory association cortex (cluster 1); bilateral somatosensory association cortex, dPCC, bilateral secondary visual cortex/BA18, and bilateral associative visual cortex/BA19 (cluster 2); and also left fusiform gyrus, left superior temporal gyrus, left middle temporal gyrus, left supramarginal gyrus, and left associative visual cortex (cluster 3) (Fig 6C; Table 1). The group differences reflected a pattern of negative correlations (anticorrelations) between the vmPFC seeds and these regions (all three clusters) in the Control group versus a pattern of either positive correlation in the SAD group (cluster 1 and 2, including posterior vACC; Fig 9A & 9B) or the absence of significant correlation in the SAD group (cluster 3; Fig 9C). There were no significant correlations within the SAD group between functional connectivity measures and clinical measures (LSAS, SPAI, and STAI).

## Discussion

People with SAD exhibited widespread alterations of the intrinsic functional brain organization of the reward system. Positive temporal correlations between two major components of the reward system, the NAcc and the ventral region of the vmPFC, were significantly reduced in the SAD group. Functional connectivity between both the NAcc and the vmPFC seed regions with multiple other anterior brain regions were significantly reduced in SAD, including prefrontal and anterior cingulate regions associated with decision-making. Conversely, the SAD group showed significantly increased functional connectivity between both the NAcc and the vmPFC seed regions with multiple posterior brain regions, including posterior ACC and posterior parietal association cortex. In these posterior regions, the Control group exhibited significant negative correlations (anticorrelations) with NAcc and vmPFC, whereas the SAD group exhibited either a reversal with a positive correlation or the absence of a correlation. Thus, the SAD group exhibited markedly decreased functional connectivity both between reward regions and also between reward regions and lateral prefrontal cortex, and markedly increased functional connectivity between reward regions and more posterior brain regions.

The SAD group showed greatly reduced functional connectivity within major components of the reward system, namely the NAcc and the vmPFC. The NAcc is associated with reward anticipation [4,5] and the vmPFC is associated with encoding the value of reward [8]. If social interaction is typically a source of substantial reward, then the absence of such reward due to chronic fear and anxiety about social interaction in SAD may diminish the frequency of interactions among these components of the reward system. Alternatively, this reduced functional connectivity among these components of the reward system may result in people with SAD seeking less social interaction. The core reward system was also significantly less correlated in the SAD group with other brain regions implicated in decision-making and cognitive control that often interact with reward regions. These regions included bilateral DLPFC, bilateral lateral anterior prefrontal cortex/BA10, bilateral inferior frontal gyrus, and anterior regions of the dorsal anterior cingulate cortex (dACC), and subgenual ACC.

Whereas functional connectivity was decreased within the reward system and between the reward system and anterior brain regions, functional connectivity was increased in the SAD group between the reward regions and multiple posterior brain regions. There were also contrasting patterns in the group differences in anterior and posterior regions. In anterior regions, the SAD group exhibited positive correlations between reward regions, but these correlations were significantly less than in the Control group. In posterior regions, the Control group exhibited negative correlations with reward regions, whereas the SAD group exhibited either a positive correlation or the absence of a correlation; in both cases the SAD group exhibited significantly greater correlations relative to the Control group.

More specifically, the SAD group exhibited a positive correlation (and not the negative correlation of the Control group), between both the NAcc and the vmPFC and posterior regions including the posterior regions of the ACC and posterior parietal cortex. The findings in the Control group were consistent with a previous report of anticorrelated networks between the posterior ACC and both the striatum and vmPFC [27]. Posterior ACC has been shown to be involved in reward-related decisions, becoming more activated as punishment increased [28] or during decisions that involve minimizing punishment [29]. The hyperconnectivity and lack of anticorrelations between reward regions and posterior ACC may reflect changes in the network due to the experience of avoiding social punishment and evaluation. Again, an alternative is that this hyperconnectivity may result in seeking less social interaction to avoid social punishment and evaluation. This, together with the decreased connectivity between the NAcc and vmPFC, may reflect an altered reward network in which reward prediction regions are less correlated with value encoding regions, and both of these reward regions are more correlated with regions that involve punishment. This may help explain how altered brain networks contribute to socially avoidant behavior in people with SAD.

Previous neuroimaging studies in SAD have found reduced structural connectivity (white-matter organization) and resting-state functional connectivity between subcortical regions and orbitofrontal cortex [30–33]. The reduced relations between prefrontal and subcortical regions, such as the amygdala, could reflect decreased prefrontal regulation of subcortical regions [34]. The present findings of reduced functional connectivity between prefrontal and subcortical reward-related regions also suggest reduced regulation of social reward.

There are both psychological and technical limitations of the present study. For the SAD group, the social interactions with researchers involved in performing the imaging may have influenced the nature of thought processes reflected in the connectivity measures. Also, there were no significant correlations between measures of SAD severity (LSAS, SPAI, STAI) and functional connectivity measures. Future research could explore the relation of reduced functional connectivity between the NAcc and vmPFC in SAD patients with tasks that involve social reward. A technical limitation of the study is that we acquired data with a relatively long time of repetition (TR = 6 seconds). We used the 6 second TR in order to achieve high spatial resolution with whole-brain coverage because previous research has demonstrated that array coils provide the biggest increases in temporal SNR at high spatial resolutions [35]. Although this TR is unusually long, there is evidence that there are no significant differences in the correlation strengths between the resting state networks when comparing TRs of 2.5 s and 5 s [36]. Furthermore, the correlations observed in the Control group were similar to prior studies using more conventional shorter TRs [37, 38].

We investigated the intrinsic functional connectivity of the human reward system in people with social anxiety disorder. People with SAD had decreased functional connectivity between the NAcc seed, which encodes reward anticipation, and the vmPFC, which encodes reward value. People with SAD also had decreased functional connectivity between the vmPFC seed and the lateral anterior prefrontal cortex and the DLPFC, which are associated with decision making. The SAD group also did not have the typical anticorrelations between both the NAcc seed and the vmPFC seed with the posterior ACC, a region that is associated with punishment [29]. The observation that independent analyses of NAcc and vmPFC seeds yielded similar differences in connectivity between the SAD and Control groups suggests that there is a widespread difference in reward-network functional connectivity in SAD, spanning regions involved in both reward anticipation and reward receipt and valuation.

## Supporting Information

S1 Fig Nucleus Accumbens Seeds Functional Connectivity Network of Control > SAD. Resting-state connectivity for **(A)** left nucleus accumbens (MNI coordinates) (-8,12,1), and **(B)** right nucleus accumbens (MNI coordinates) (11,11,1) for control group > SAD (Cluster-wise FDR corrected, p < .05).
(DOCX)

S2 Fig Nucleus Accumbens Seeds Functional Connectivity Network of SAD > Control. Resting-state connectivity for **(A)** left nucleus accumbens (MNI coordinates) (-8,12,1), and **(B)** right nucleus accumbens (MNI coordinates) (11,11,1) for SAD > Control group (Cluster-wise FDR corrected, p < .05).
(DOCX)

S3 Fig Ventromedial Prefrontal Cortex Seed Functional Connectivity Network of Control > SAD. Resting-state connectivity for ventromedial prefrontal cortex **(A)** seed 1 (MNI coordinates) (-6, 24, -21), **(B)** seed 2 (MNI coordinates) (6, 30, -9), and **(C)** seed 3 (MNI coordinates) (9, 27, -12) for SAD > Control group (Cluster-wise FDR corrected, p < .05).
(DOCX)

S4 Fig Ventromedial Prefrontal Cortex Seed Functional Connectivity Network of SAD > Control. Resting-state connectivity for ventromedial prefrontal cortex **(A)** seed 1 (MNI coordinates) (-6, 24, -21), **(B)** seed 2 (MNI coordinates) (6, 30, -9), and **(C)** seed 3 (MNI coordinates) (9, 27, -12) for Control group > SAD (Cluster-wise FDR corrected, p < .05).
(DOCX)
