# Supplementary material for: Altered Resting-State Functional Connectivity of the Frontal-Striatal Reward System in Social Anxiety Disorder
Source: PLoS One. 2015 Apr 30;10(4):e0125286. doi: 10.1371/journal.pone.0125286 (PMC4416052; doi:10.1371/journal.pone.0125286)

**Figure S1: Nucleus Accumbens Seeds Functional Connectivity Network of Control > SAD.** Resting-state connectivity for **(A)** left nucleus accumbens (MNI coordinates)

(-8,12,1), and **(B)** right nucleus accumbens (MNI coordinates) (11,11,1) for control group > SAD (Cluster-wise FDR corrected, p < .05).


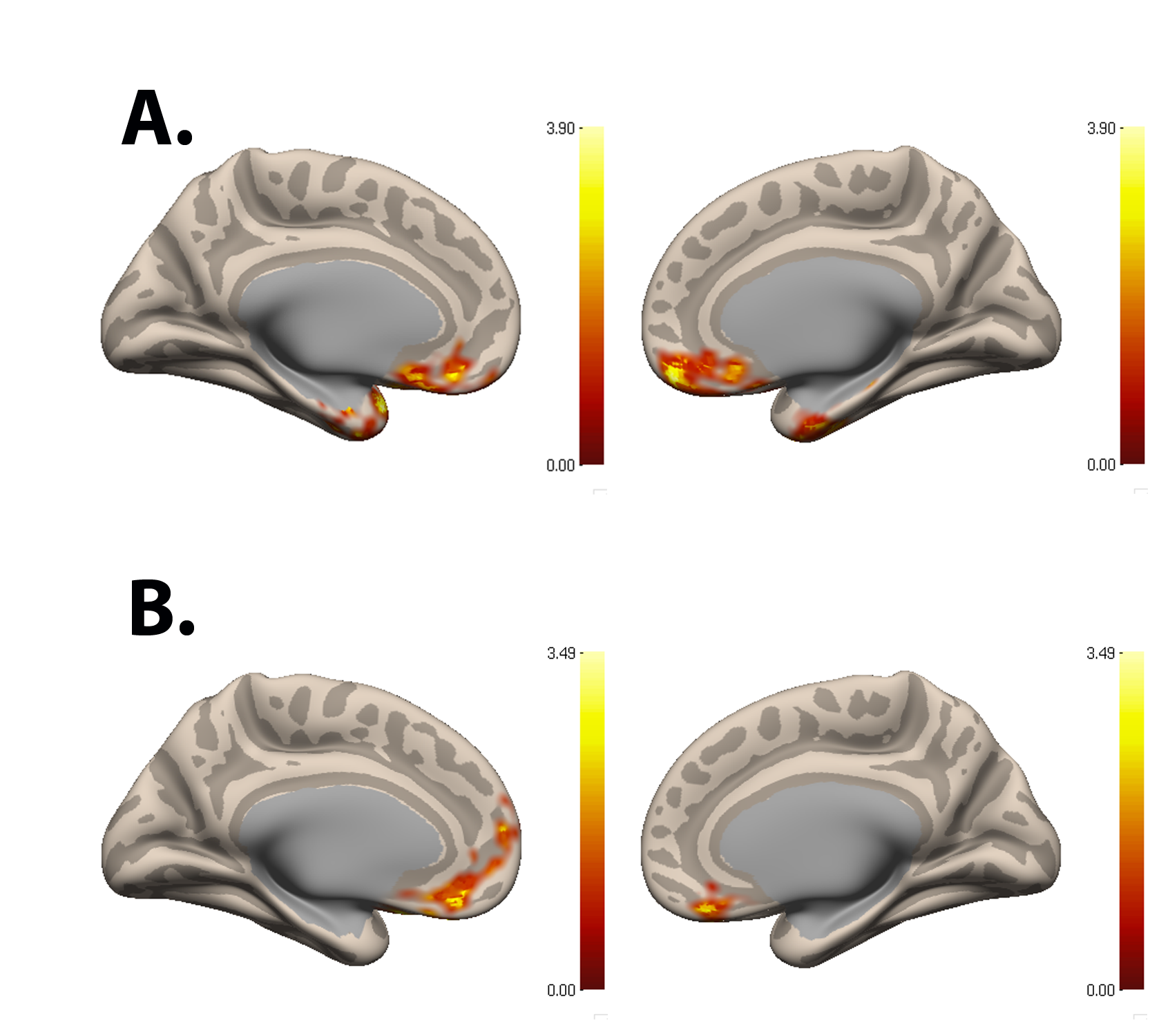

Supplement: S1 Fig — Resting-state connectivity for (A) left nucleus accumbens (MNI coordinates) (-8,12,1), and (B) right nucleus accumbens (MNI coordinates) (11,11,1) for control group > SAD (Cluster-wise FDR corrected, p < .05). (DOCX) [file pone.0125286.s001.docx]
