# Supplementary material for: Altered Resting-State Functional Connectivity of the Frontal-Striatal Reward System in Social Anxiety Disorder
Source: PLoS One. 2015 Apr 30;10(4):e0125286. doi: 10.1371/journal.pone.0125286 (PMC4416052; doi:10.1371/journal.pone.0125286)

**Figure S4: Ventromedial Prefrontal Cortex Seed Functional Connectivity Network of SAD > Control.** Resting-state connectivity for ventromedial prefrontal cortex **(A)** seed 1 (MNI coordinates) (-6, 24, -21), **(B)** seed 2 (MNI coordinates) (6, 30, -9), and **(C)** seed 3 (MNI coordinates) (9, 27, -12) for Control group > SAD (Cluster-wise FDR corrected, p < .05).


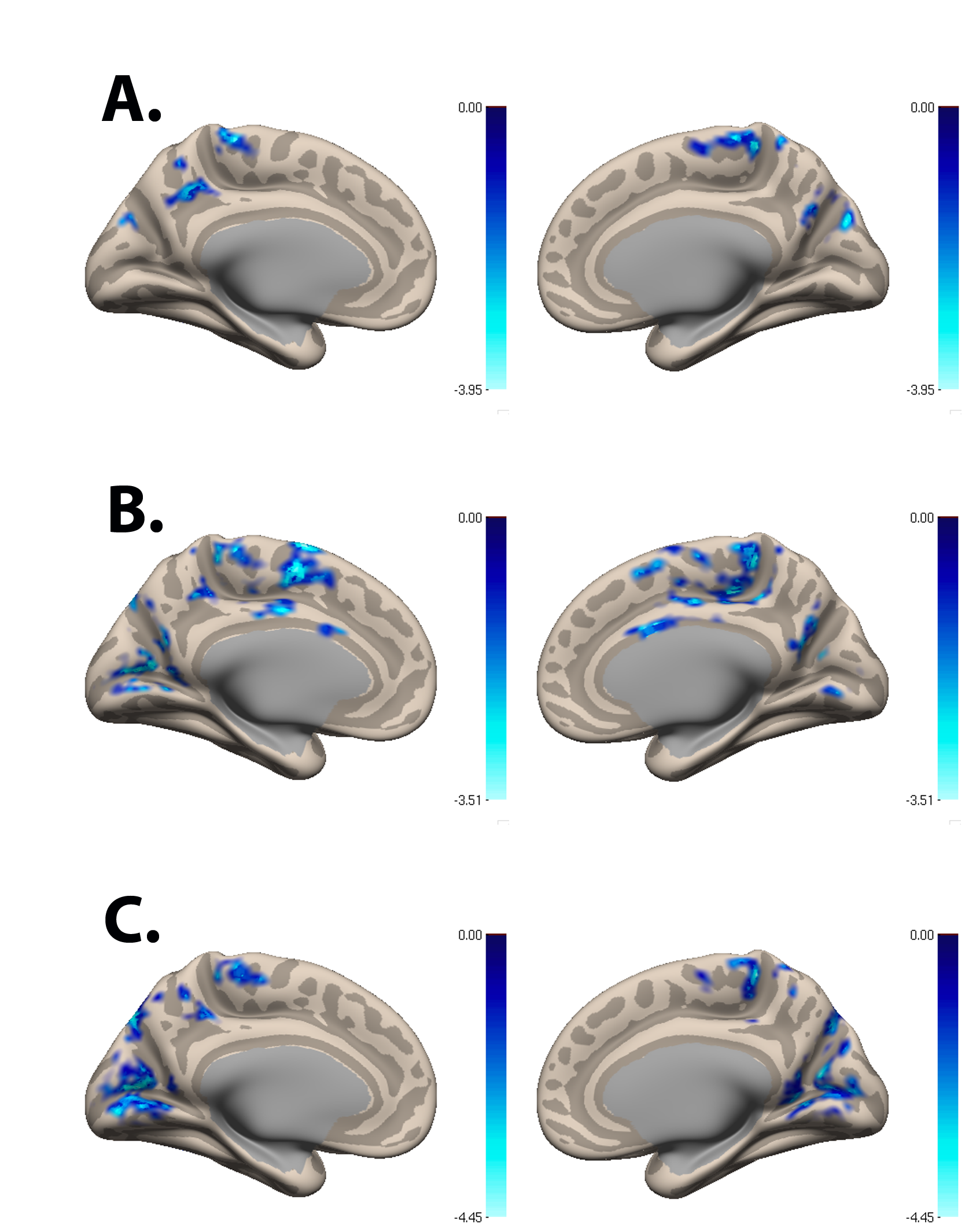

Supplement: S4 Fig — Resting-state connectivity for ventromedial prefrontal cortex (A) seed 1 (MNI coordinates) (-6, 24, -21), (B) seed 2 (MNI coordinates) (6, 30, -9), and (C) seed 3 (MNI coordinates) (9, 27, -12) for Control group > SAD (Cluster-wise FDR corrected, p < .05). (DOCX) [file pone.0125286.s004.docx]
